# Correction: Aggressive Chemotherapy and the Selection of Drug Resistant Pathogens

**DOI:** 10.1371/journal.ppat.1005301

**Published:** 2015-11-17

**Authors:** The PLOS Pathogens Staff

## Notice of Republication

This article was republished on October 19, 2015, to correct errors in equations that were introduced during the typesetting process. Specifically, the exponent in the fourth equation in the Material and Methods should be -8.821, not 8.821, and this has been corrected. The publisher apologizes for the errors. Please download this article again to view the correct version. The originally published, uncorrected article and the republished, corrected article are provided here for reference.

## Supporting Information

S1 File Originally published, uncorrected article. (PDF)

S2 File Republished, corrected article. (PDF)
