# Supplementary material for: Correction: Aggressive Chemotherapy and the Selection of Drug Resistant Pathogens
Source: PLoS Pathog. 2015 Nov 17;11(11):e1005301. doi: 10.1371/journal.ppat.1005301 (PMC4648503; doi:10.1371/journal.ppat.1005301)
Supplement: S2 File — (PDF) [file ppat.1005301.s002.pdf]

PLOS | PATHOGENS

Aggressive Chemotherapy and the Selection of Drug Resistant Pathogens

Silvie Huijben[1¤a*], Andrew S. Bell[1], Derek G. Sim[1], Danielle Tomasello[1¤b], Nicole Mideo[1], Troy Day[2], Andrew F. Read[1,3*]

1 Center for Infectious Disease Dynamics, Departments of Biology and Entomology, Pennsylvania State University, University Park, Pennsylvania, United States of America, **2** Departments of Mathematics, Statistics and Biology, Jeffery Hall, Queen's University, Kingston, Ontario, Canada, **3** Fogarty International Center, National Institutes of Health, Bethesda, Maryland, United States of America

Abstract

Drug resistant pathogens are one of the key public health challenges of the 21st century. There is a widespread belief that resistance is best managed by using drugs to rapidly eliminate target pathogens from patients so as to minimize the probability that pathogens acquire resistance *de novo*. Yet strong drug pressure imposes intense selection in favor of resistance through alleviation of competition with wild-type populations. Aggressive chemotherapy thus generates opposing evolutionary forces which together determine the rate of drug resistance emergence. Identifying treatment regimens which best retard resistance evolution while maximizing health gains and minimizing disease transmission requires empirical analysis of resistance evolution *in vivo* in conjunction with measures of clinical outcomes and infectiousness. Using rodent malaria in laboratory mice, we found that less aggressive chemotherapeutic regimens substantially reduced the probability of onward transmission of resistance (by >150-fold), without compromising health outcomes. Our experiments suggest that there may be cases where resistance evolution can be managed more effectively with treatment regimens other than those which reduce pathogen burdens as fast as possible.

Citation: Huijben S, Bell AS, Sim DG, Tomasello D, Mideo N, et al. (2013) Aggressive Chemotherapy and the Selection of Drug Resistant Pathogens. PLoS Pathog 9(9): e1003578. doi:10.1371/journal.ppat.1003578

Editor: L. David Sibley, Washington University School of Medicine, United States of America

Received March 18, 2013; **Accepted** July 10, 2013; **Published** September 12, 2013

Copyright: © 2013 Huijben et al. This is an open-access article distributed under the terms of the Creative Commons Attribution License, which permits unrestricted use, distribution, and reproduction in any medium, provided the original author and source are credited.

Funding: This work was supported by the National Institute of General Medical Sciences (R01GM089932). The funders had no role in study design, data collection and analysis, decision to publish, or preparation of the manuscript.

Competing Interests: The authors have declared that no competing interests exist.

* E-mail: silviehuijben@gmail.com (SH); a.read@psu.edu (AFR)

¤a Current address: CRESIB - Barcelona Centre for International Health Research, Barcelona, Spain.
¤b Current address: Neuroscience Program, The State University of New York at Buffalo, Buffalo, New York, United States of America.

Introduction

At the end of his 1945 Nobel Prize lecture, Alexander Fleming warned of the dangers of drug resistance and proclaimed that "if you use penicillin, use enough" [1]. Only by killing all bacteria in an infection, he asserted, could drug resistance be prevented. Today, this remains standard thinking [2–12]. The philosophy is that aggressive chemotherapy minimizes the probability that pathogens acquire resistance by *de novo* mutations or lateral transfer of genetic material: dead bugs can't evolve. It is why physicians frequently exhort patients to finish drug courses even after they no longer feel sick [13].

Yet the rate of adaptive evolution is determined by the available genetic variation *and* the strength of selection [14]. This means that the rate of spread of resistance alleles within a host or within a host population is a function not only of the rate at which resistance alleles arise but also of the strength of selection acting on them. This selection acts both within the host when a genetic event conferring resistance first occurs, and then subsequently as resistance spreads in a host population. And all else being equal, increasing drug pressure will increase the strength of selection. Consequently, aggressive treatment regimens - those aimed at eliminating all pathogens as fast as possible by, for instance, sufficiently high drug dose or long treatment duration - are a double-edged sword for resistance management [15]. Aggressive chemotherapy can retard the evolution of resistance by reducing pathogen population sizes and hence the chances of high-level resistance arising *de novo*. But in an infection which already contains drug-resistant pathogens, either by *de novo* mutation, lateral transfer, or by transmission from other hosts, aggressive chemotherapy will rapidly eliminate drug-susceptible competitors, thus powering the very evolution it is designed to inhibit.

Quite how these opposing evolutionary forces combine to affect the rate of resistance evolution in any particular host-parasite system is unclear. Yet without understanding that, it is impossible to determine whether Fleming's rule (or others, like 'hit hard and hit fast' [7]) are in fact sound resistance management strategies. This is particularly critical where toxicity or cost considerations place upper bounds on how much drug pressure can be applied, or where high level resistance is frequently acquired, either *de novo* or from other people. The question then becomes: among the wide range of drug doses, inter-dose intervals and treatment durations that can achieve the required clinical outcomes, which patient treatment regimen best retards the evolution of resistance? Here we present the first empirical data that shows that these need not be treatment regimens which remove susceptible pathogens as fast as possible.

Author Summary

Drug-resistance is a major public health problem. Conventional wisdom on resistance management is to use aggressive chemotherapy to kill pathogens as rapidly as possible so as to prevent them from acquiring resistance. This is the reason why physicians frequently exhort patients to finish drug courses even after they no longer feel sick. However, this approach is based on the notion that we need only prevent new resistant mutants from arising. We hypothesize that in the situation where such mutants are already present at the time of treatment, more aggressive chemotherapy will select for these the fastest by rapidly killing all sensitive competitors. Here we demonstrate in a rodent malaria model that such selection indeed occurs more intensely following aggressive treatment than following less aggressive treatment, without any benefit to host health or infectivity. This suggests that aggressive chemotherapy will not be the best way to retard resistance evolution in some - perhaps many - circumstances. We suggest that an evidence-based approach across a wide range of infectious diseases is needed to manage resistance evolution.

The reasoning is as follows. Resistant strains generally reach appreciable densities in infected patients only once drug treatment is employed. This implies that resistant pathogens are competitively suppressed by susceptible pathogens in the absence of drug treatment, and that the removal of susceptible pathogens by chemotherapy causes resistant pathogen populations to expand, a process we have termed 'competitive release', borrowing from the ecological literature [16]. We define competition very broadly to mean any negative effect of the presence of susceptible pathogens on the population of resistant pathogens; other authors have called this 'clonal interference' [17,18]. Competition could be resource-based exploitation competition, interference competition, or immune-mediated apparent competition [19]. Competitive release can generate very substantial relative and absolute fitness gains for resistant pathogens [20–25]. In acute rodent malaria infections, for example, competitive release can lead to greatly enhanced transmission of resistant parasites [16,26–28]. These data suggest that resistance might be better managed by using 'lighter touch' treatment regimens, regimens which do not clear pathogens as fast as possible; in effect, maintaining susceptible parasites in an infection for longer to suppress resistant populations [15,27–29].

For this hypothesis to be viable, there are four requirements. First, from the resistance management perspective, 1) reducing drug pressure must reduce the extent of competitive release of resistant parasites, with the consequence that, 2) less aggressive treatment reduces the onward transmission of resistant pathogens. However, resistant management strategies are only of interest if they also achieve clinical and public health gains. Therefore, we would also require that 3) less aggressive treatment regimens do not increase host infectiousness, and 4) that less aggressive treatment regimens generate improvements in host health which are as good or better than those generated by aggressive chemotherapy.

So far as we are aware, these four requirements have never been tested simultaneously for any infectious agent *in vivo*. Here we report such a test using *Plasmodium chabaudi* in laboratory mice. The results show that in this biological model, lighter touch regimens better contain already existing resistant parasites than aggressive treatment, without compromising host health.

Results

We inoculated mice with a mixture of drug-resistant and drug-susceptible parasites, whereby resistant parasites were numerically dominated by the susceptible ones, as would occur in the initial spreading phase of resistant mutants. In experiment 1, a ratio of 10^1:10^6 resistant to susceptible parasites was administered. To mimic the rarity of a *de novo* mutational event, a ratio of close to 10^1:10^9 was established in experiment 2 by seeding ~25 resistant parasites at peak susceptible parasitaemia (Fig. 1, Table 1). We then treated these infections with the anti-malarial drug pyrimethamine on day 6 post-infection (PI) with either an aggressive treatment regimen (8 mg/kg for 5 or 7 days) or 'lighter touch' treatment regimens (on just the first day of treatment, that same daily dose, or half of it, regimens we call 'moderate' and 'light' respectively, see Table 1). Lighter touch regimens we chose to test were derived from analysis of a mathematical model of within-host parasite dynamics.

When infections containing drug-resistant and drug-susceptible parasites were untreated, resistant parasites barely grew to densities above PCR detection threshold (Fig. 1AB). In fact, when resistant parasites were at densities close to *de novo* mutations, they were almost never detected in untreated infections (Fig. 1B). With increasing drug pressure, both the likelihood and the intensity of relapsing resistant parasites increased, so there were more parasites overall (Fig. 1C–J, Figs. S1, S2, S3, S4, S5; *mean parasite density*, Exp 1: $F_{2,16} = 15$, $p<0.001$, Exp 2: $F_{2,25} = 29$, $p<0.001$). As expected, parasite densities of the susceptible parasites were inversely related to the drug pressure (Fig. S6). Thus, our data show that competitive release is a positive function of drug pressure (requirement 1).

No resistant transmission stages were detected in untreated infections (except in one sample from one mouse in one experiment; Fig. 2AB, Figs. S3,S5). Increased drug pressure increased the duration of the infectious period for resistant parasites (Fig. 2C–H; *Number of gametocyte-positive days*, Exp 1: $F_{2,16} = 6.0$, $p = 0.011$, Exp 2: $F_{2,25} = 21$, $p<0.001$) as well as the density of resistant transmission stages (Fig. 2IJ). Consequently, the probability that resistant parasites would be transmitted increased with drug pressure (Fig. 3, *probability of resistant transmission*, Exp 1: $F_{2,16} = 7.3$, $p = 0.006$, Exp 2: $F_{2,25} = 34$, $p<0.001$). The probability of resistant parasites infecting mosquitoes was negligible following light touch treatment and increased 162-fold following aggressive treatment (Fig. 3AB). Hence, the probability of onward transmission of resistant parasites is a positive function of drug pressure (requirement 2).

Aggressively treated infections were also the most infectious overall as there were more asexual parasites to produce transmission stages (Fig. 3CD, Fig. S7; *probability of overall transmission*, Exp 1: $F_{3,22} = 4.8$, $p = 0.010$; Exp 2: $F_{3,31} = 30$, $p<0.001$). Susceptible parasites did not contribute substantially to the overall post-treatment infectiousness in the higher dose treatments, but dominated in the light touch treatment and untreated infections (Figs. S2, S3, S4, S5, S6, S7, S8). Thus, overall infectiousness (transmission of parasites of either strain) was also a positive function of drug pressure (requirement 3).

Mice that did not receive drug treatment were the most anemic and lost the most body mass compared to drug-treated infections. However, more aggressive drug treatment did not result in more favorable host health outcomes compared to lighter touch treatment (Fig. 4). In experiment 1, aggressive treatment caused a higher body weight loss and anemia during the acute phase possibly due to drug toxicity. Additionally, aggressive treatment had a negative downstream effect by causing a prominent

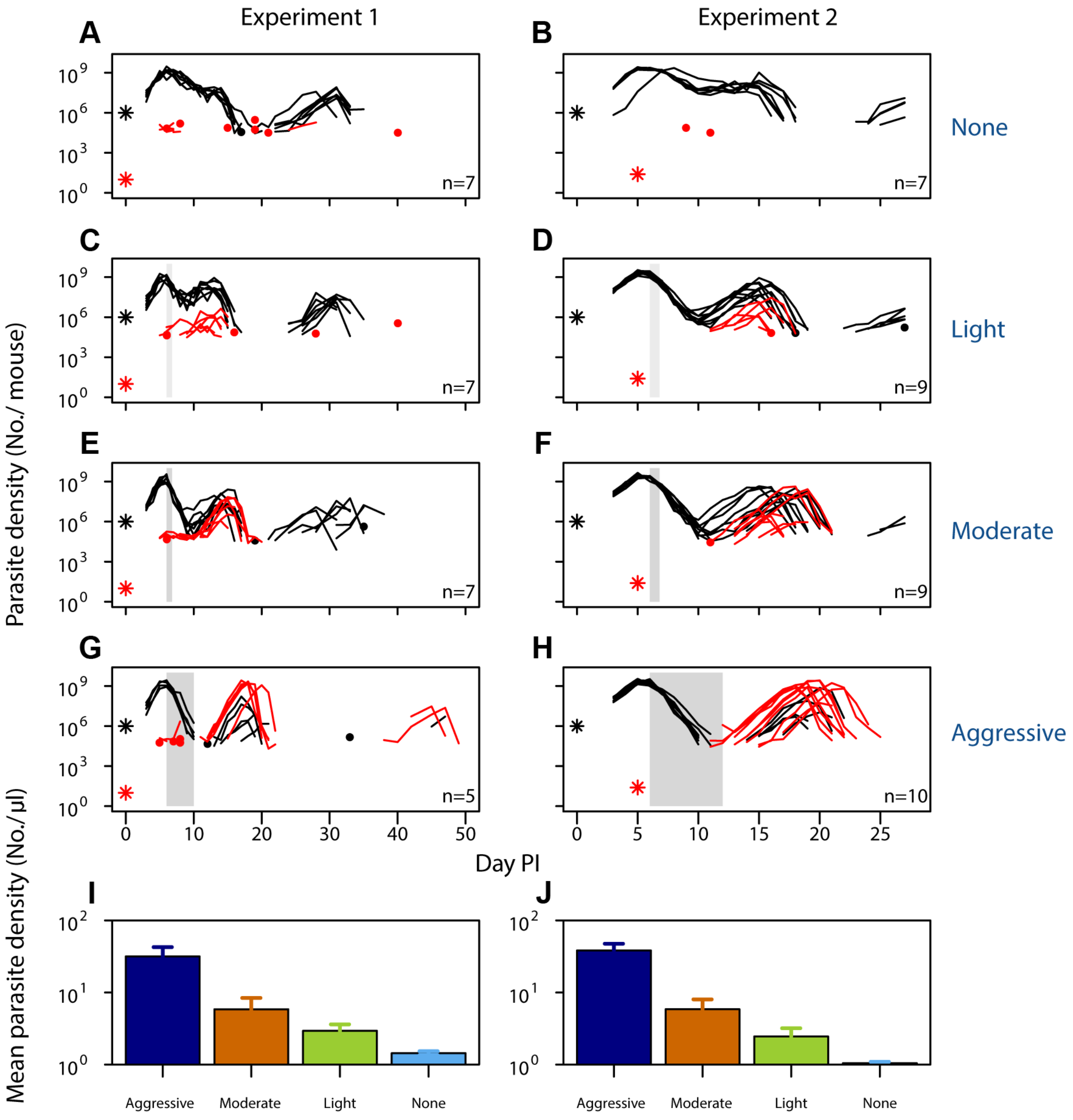

Figure 1. Selection of resistant parasites increases with drug pressure. Parasite dynamics (top four rows) of individual mice in mixed infections of resistant (red lines) and susceptible (black lines) parasites in experiment 1 (left panels) and experiment 2 (right panels) in infections that received no (A,B), light (C,D), moderate (E,F) and aggressive (G,H) treatment. Asterisks indicate the number of parasites at inoculation, dots indicate positive counts detected in individual mice on a single day (parasites not detected the day before and after), grey bars show timing and duration treatment. Bottom row: geometric mean asexual parasite density of resistant parasites during the measured infection period (day 3–49 in experiment 1 [I] and day 3–28 PI in experiment 2 [J]). Data are means (± s.e.m.) with sample sizes (n, number of mice) as in subplots.
doi:10.1371/journal.ppat.1003578.g001

secondary bout of anemia caused by relapsing resistant parasites. Such secondary anemia bouts were to a lesser extent also observed in some of the other drug-treated groups (Fig. 4, top panels). Thus, less aggressive treatment regimens generated health outcomes at least as good as those generated by aggressive chemotherapy (requirement 4).

Discussion

The stronger the strength of selection, the more rapid is the spread of a favored allele. This means that, all else equal, reducing drug use will slow the evolution of resistance in a population, thus prolonging the useful lifespan of a drug. This is why over-the-counter antibiotic sales are banned in many countries, why there are numerous policies aimed at ensuring drugs are used only when the target pathogen is present, and why there are calls to remove antibiotics from animal feed [3,30]. Our experiments show the same thinking can apply within hosts. We found that increasing drug pressure *in vivo* led to larger populations of resistant parasites with an increased likelihood of onward transmission. Lighter touch regimens gave better resistance management, consistent with the hypothesis that when resistant pathogens are present, aggressive chemotherapy need not best manage resistance evolution. Given this, we suggest that the use of aggressive chemotherapy needs very careful justification.

The most obvious justification is patient need: in many cases, host health improves more rapidly with increasingly aggressive chemotherapy. But at least in our animal model, these gains can

Table 1. Experimental setup of mixed infection of experiments 1 and 2.

	Treatment	Treatment duration (days)	Dose	Clone R:S ratio	Mice inoculated (in analysis)
Exp. 1	Untreated	-	-	10^1:10^6	8(7)
	Light	1	4 mg/kg	10^1:10^6	8(7)
	Moderate	1	8 mg/kg	10^1:10^6	8(7)
	Aggressive	5	8 mg/kg	10^1:10^6	8(5)
Exp. 2	Untreated	-	-	$\sim 10^1$:10^9	10 (7)
	Light	1	4 mg/kg	$\sim 10^1$:10^9	10 (9)
	Moderate	1	8 mg/kg	$\sim 10^1$:10^9	10(9)
	Aggressive	7	8 mg/kg	$\sim 10^1$:10^9	10(10)

Infections were initiated with a mixture of resistant and susceptible parasites with the specified R:S ratio inoculum on day 0 (Experiment 1) or with 10^6 susceptible parasites on day 0 and ~25 resistant parasites on day 5 (Experiment 2). Number of mice at the start of the experiment is given, with the number used in the analysis (after exclusion based on infection establishment and mortality; see Materials and Methods for details) in brackets.
doi:10.1371/journal.ppat.1003578.t001

rapidly saturate. Aggressive treatment generated additional selection for resistance without additional health gains or additional reductions in infectiousness (Figs. 1–4). In clinical trials, shorter antibiotic regimens can be as effective as long-course regimens [31–37]. There is clearly scope in at least some cases for using lighter touch chemotherapy without compromising patient or public health. Where clinical requirements do demand aggressive chemotherapy, evolutionary mismanagement may be an unavoidable side effect. In such situations, it may be critical to use alternate strategies such as combination therapy and transmission-blocking measures to retard the resulting resistance evolution.

There are also circumstances where aggressive chemotherapy can be justified as a resistance management strategy. This is Fleming's thinking: use enough drug pressure to eliminate mutational inputs into the system, thus reducing the probability resistance will emerge in a patient. This can work but it is a risky gambit as the reductions in the probability of resistance arising *de novo* accrue only by imposing extremely potent selection for the very resistance it is intended to prevent (Figs. 1–2). In our experiments, aggressive treatment was very effective at finding parasites with high level resistance when they were very rare, indeed close to the frequency of a *de novo* mutational event, and getting them to densities which were both transmissible and detrimental to host health (Figs. 1–4).

Fleming's advice is unambiguously sound only in situations which are not the main challenge for resistance management. If sufficient drug pressure can be applied to eliminate all pathogens, wild-types and mutants, resistance evolution can be prevented [7,10,38–41]. But then there is no evolutionary problem to solve: the drug will work forever provided that adequate doses are always administered. The resistance management challenge arises only when the realities of toxicity and cost impose upper bounds on the drug pressure that can be brought to bear. It is the pathogens which can survive those upper bounds—those with high level resistance—which undermine the therapeutic utility of a drug, and can render it clinically useless. Once there is a finite probability that parasites can acquire high-level resistance, Fleming's dogma may or may not be correct. This is so even if aggressive chemotherapy successfully kills pathogens with intermediate levels of resistance. Removing these 'mutational stepping stones' can decrease the probability that high level resistance will arise *de novo*, but unless that probability is zero, aggressive chemotherapy can reduce or enhance the probability of full resistance emerging. Again, this is because the probability of resistance emergence is a function of both the probability that a genetic event conferring high level resistance occurs *and* the strength of selection acting on any which do.

The best way to prevent resistance emergence depends on the relative magnitude of both these forces. Estimating those is a non-trivial problem in applied evolutionary biology, not least because the nature of the within-host ecology and the probability of acquiring resistance by mutational or lateral gene transfer will be very disease and context-specific—and hard to estimate before high-level resistance arises. Worse, the relevant probabilities are not static. Even when aggressive chemotherapy does minimize the probability *de novo* resistance occurs, it may become less than optimal for individual patients once high level resistance is established and spreading in the population. For instance, in Estonia and Cuba, more than 90% of patients acquire their drug resistant TB from the community [42]. Similarly, chloroquine became ineffective against malaria in Africa not as a result of frequent *de novo* resistant mutations, but because the highly resistant progeny of an Asian parasite spread across the entire African continent [43,44]. In such cases, it is unclear that continuing to treat patients as if *de novo* resistance is the main threat is best either for the patient or for slowing the evolution which undermines the utility of the drugs involved.

Our data suggest that there may be situations where resistance evolution can be managed more effectively with treatment regimens other than those which reduce pathogen burdens as fast as possible. A related argument has been made in the context of resistance management in cancer [45–47]. The same logic also applies when choosing drugs for multidrug treatments against both cancer and infections. Drug combinations can be synergistic or antagonistic depending on whether pathogen inhibition is more or less inhibitory than the component drugs. Synergistic combinations are in effect more aggressive treatment, and at least against bacteria *in vitro*, these accelerate the evolution of resistance [48,49].

We think those data, and the data we report here, demonstrate the need for an evidence-based approach to identifying patient treatment regimens which best manage resistance management without compromising clinical outcomes. As we have emphasized elsewhere [15], the appropriate thing to do will almost certainly vary, likely depending among other things on the drug, the pathogen, the host, clinical need, the pharmacokinetics involved, the phenotypic response of pathogens to chemotherapy, the efficacy of natural immunity, and the probability that high level

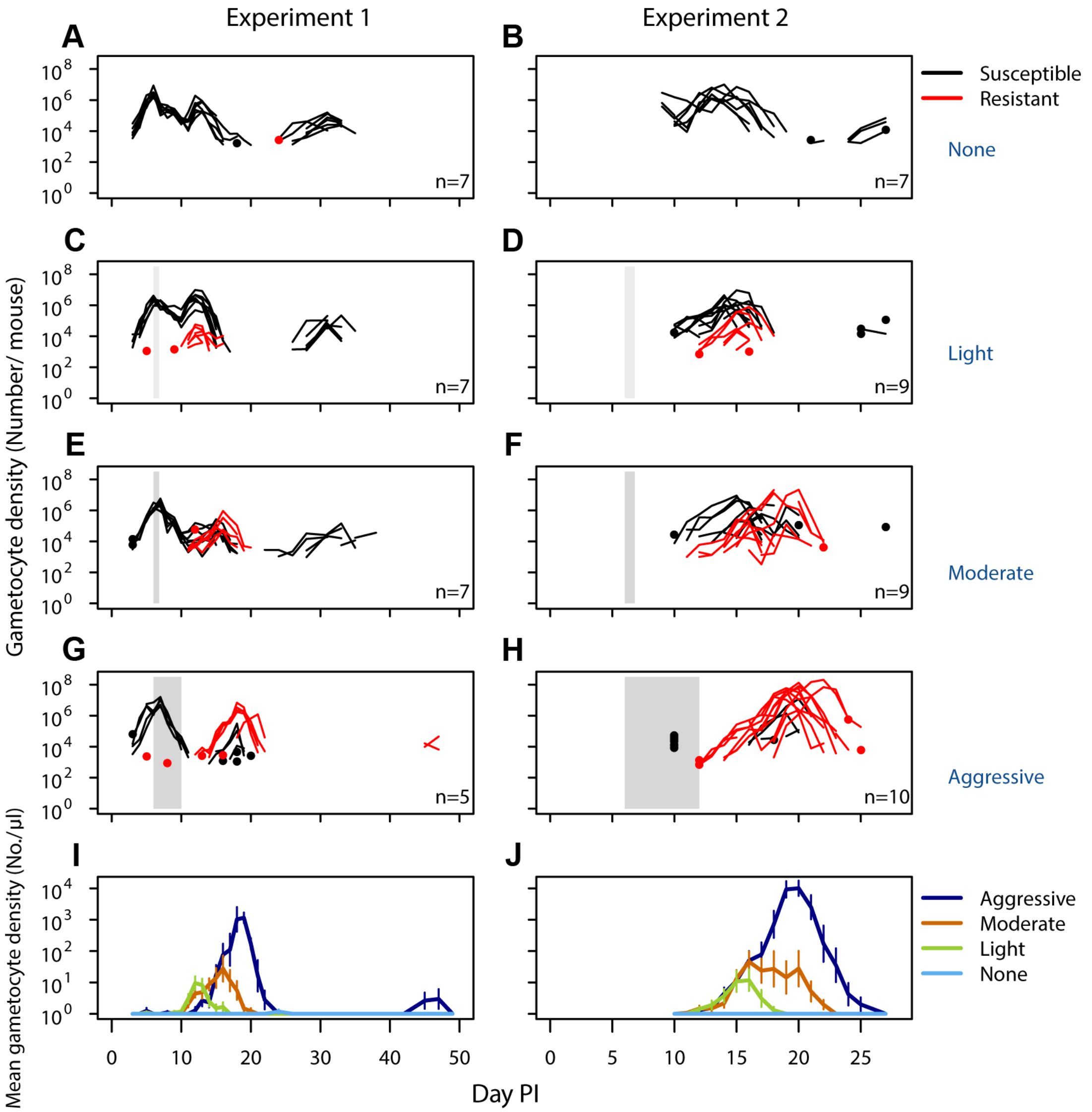

Figure 2. Density of resistant transmission stages increases with drug pressure. Gametocyte (transmission stage) dynamics (top four rows) of individual mice in mixed infections of resistant (red lines) and susceptible (black lines) parasites in experiment 1 (left panels) and experiment 2 (right panels) in infections that received no (A,B), light (C,D), moderate (E,F) and aggressive (G,H) treatment. Dots indicate positive counts detected in individual mice on a single day, grey bars show timing and duration treatment. Bottom row: Mean gametocyte dynamics from experiment 1 (I) and experiment 2 (J) of resistant parasites for each treatment group (legend). Data are means (± s.e.m.) with sample sizes (n, number of mice) as in subplots. Note experiments 1 and 2 have a different duration of the experiment and gametocyte samples were only taken from day 10 onwards in experiment 2.
doi:10.1371/journal.ppat.1003578.g002

resistance is present before treatment starts. We will be rather surprised if there prove to be simple generalities for the resistance management challenges which arise when there are limits to how much drug pressure can be applied, and when resistance to those drug concentrations can arise. Of course, we cannot test that proposition from our experiments with one animal model and one drug, let alone make clinical recommendations. There is a large knowledge gap and a need for many more experiments which analyze *in vivo* the fate of resistant pathogens together with measures of clinical outcome and infectiousness, as we have done. Only with such data will it become possible to determine when Fleming's advice to 'use enough' best manages resistance evolution. Meanwhile, our data show that Fleming's advice essentially fights fire with fire. If it falls short for whatever reason, it can promote the very evolution it is intended to retard.

Materials and Methods

Ethics statement

The study was carried out in strict accordance with the recommendations in the guide for the Care and Use of Laboratory Animals of the National Institutes of Health. The protocol was approved by the Animal Care and Use Committee of the Pennsylvania State University (Permit Number: 35790).

Choice of drug regimens

A mathematical model was utilized in the decision process for choosing drug regimes to test experimentally. The basic model, derived from Mideo et al. [50], is of the following generalized form:

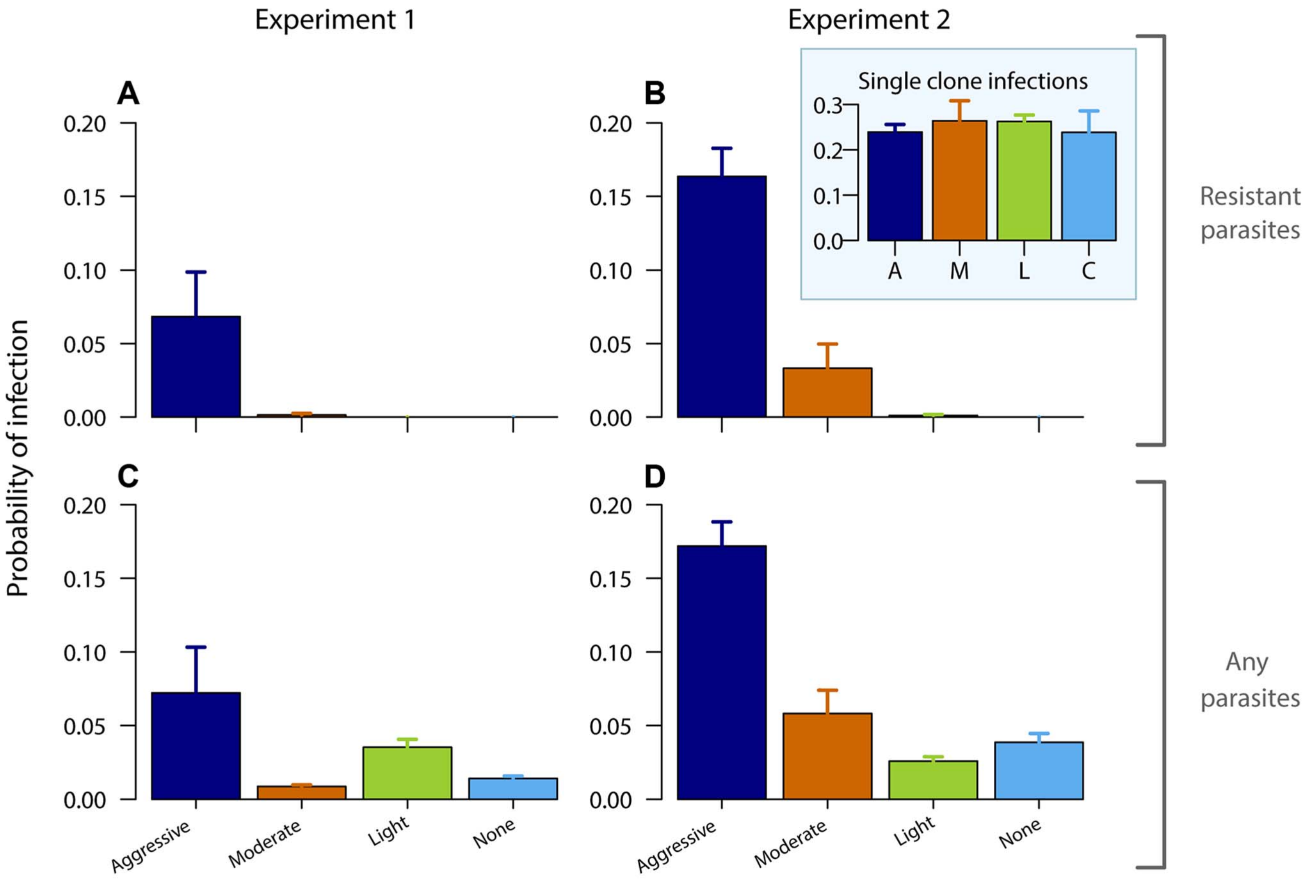

Figure 3. Aggressive chemotherapy increases infectiousness. Probability of infecting mosquitoes from resistant gametocytes (A,B) and from all gametocytes (C,D – resistant and susceptible combined) for experiment 1 (left panels) and experiment 2 (right panels) estimated from an empirically derived gametocyte density – infectivity relationship (see Materials and Methods) during the post-treatment period (day 10–28 PI) following aggressive (A), moderate (M), light (L) or no (C) treatment. Inset graph shows probability of infection of resistant parasites in absence of competition. Data are means (± s.e.m.) with sample sizes as in Table 1.

doi:10.1371/journal.ppat.1003578.g003

$$P_S(t+1)=f_S(P_S(t),\,N(t))$$

$$P_R(t+1)=f_R(P_R(t),\,N(t))$$

$$N(t+1)=g(N(t-\tau))-h_S(P_S(t),\,N(t))-h_R(P_R(t),\,N(t))$$

where P_S, P_R and N track the daily densities of drug-susceptible parasites, drug-resistant parasites and host red blood cells. The functions f_S and f_R describe the process of red blood cell invasion by parasites as well as the production of progeny parasites within an infected cell. The functions h_S and h_R account for the loss of red blood cells due to infection. In both cases, the subscripts denote the fact that while the functions are the same, they take on unique parameter values for each parasite strain (estimated by maximum likelihood procedures described in Mideo et al. [50]). The function g describes the daily production of new red blood cells which depends on red blood cell density τ days earlier to account for the maturation time of blood cell precursors. Details of model assumptions, derivations and functional forms are in Mideo et al. [50].

Drug activity was superimposed on the model described above. Phenomenologically, anti-malarial drug action can be described as operating via a threshold mechanism – above a threshold drug concentration, a given proportion of susceptible parasites are killed and below the threshold, there is no effect of drugs [51]. The length of time that the within-host drug concentration is above this threshold and thus how long the drug-induced parasite decline continues depends on dose, dosing interval and duration of treatment. This was shown to be true for pyrimethamine against *P. chabaudi* in mice [52]. Using those data we estimated how drug dose affects the duration of 'drug activity'. The number of additional days (beyond the inoculation days) of drug activity, a, is given by

$$a=3.557-2.586\big/\left(1+e^{-8.821+b}\right)$$

where b is the drug dose in mg/kg. We also estimated that each day of drug activity results in a 94% decline in susceptible parasite numbers.

In the absence of drug treatment, the dynamics of the two parasite strains and the host red blood cells are governed by the basic competition model. In the presence of drugs (on days drugs are administered+a days after), the density of drug-susceptible parasites on the next day is a simple linear function of the current

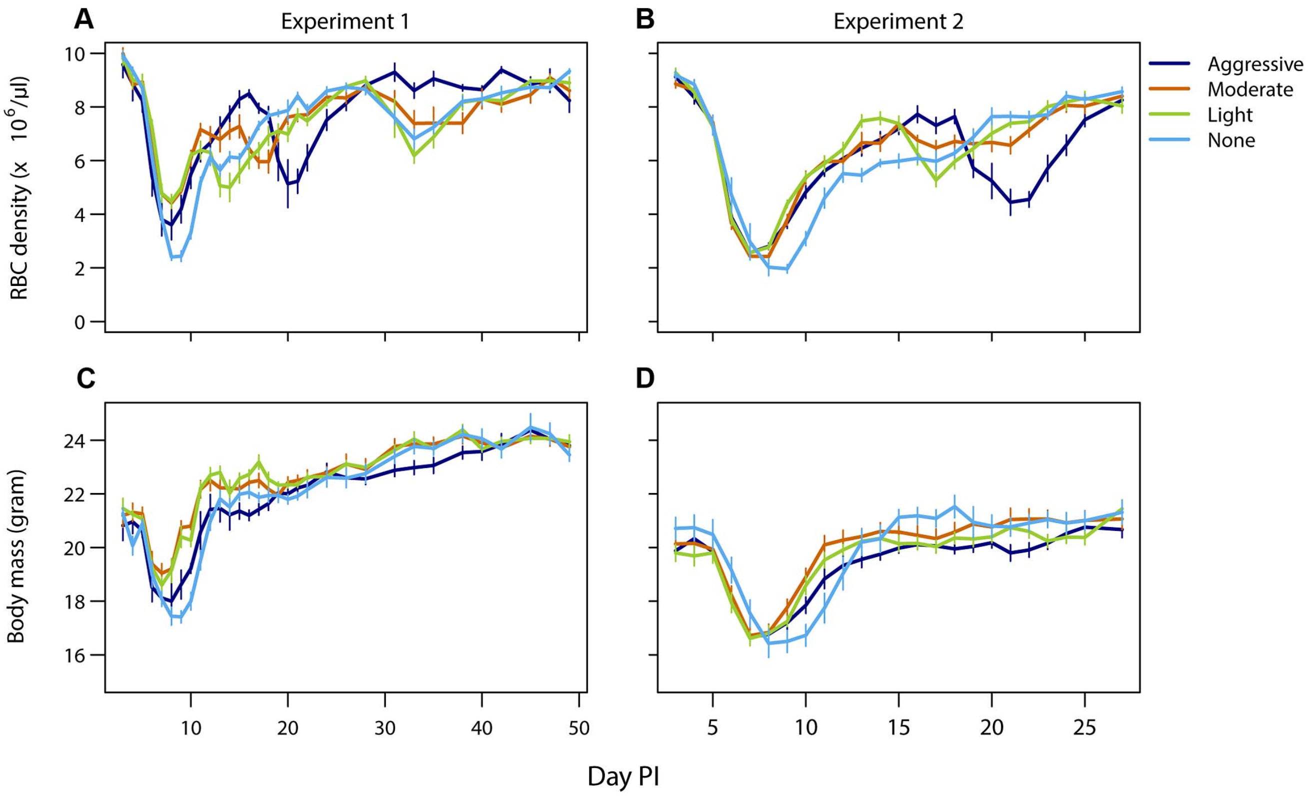

Figure 4. Light touch regimens improve host health as much or better than aggressive chemotherapy. Mean red blood cell (A,B) and body mass (C,D) dynamics of mice in experiment 1 (left panels) and experiment 2 (right panels) that were treated (day 6 PI) with an aggressive (dark blue), moderate (orange) or a light (green) drug dose or left untreated (light blue). Data are means (± s.e.m.) with sample sizes as in Table 1.
doi:10.1371/journal.ppat.1003578.g004

density. Thus, P_S $(t+1) = 0.06$ P_S (t), while the rest of the system remains unaltered. Using this approach, we were able to qualitatively capture the outcome of competition experiments between drug-susceptible and drug-resistant malaria parasite clones in the presence of drugs, with conventional drug treatment (a dose of 8 mg/kg for 4 days starting at the onset of disease symptoms) and with different initial ratios of susceptible to resistant parasites. This model was used to predict the effects of different drug regimens on the success of the drug-resistant parasite clone within a host. The treatment regimens which were predicted to result in substantially different infection dynamics and the best potential for suppressing the resistant parasites were included in the experiment (Figure S9).

Experimental methods

Two experiments (Table 1) were initiated with *Plasmodium chabaudi* drug-susceptible clone AJ_{5p} and pyrimethamine-resistant clone $AS_{6p(pyr1A)}$ in approximately 8 week old female C57Bl/6 laboratory mice (Charles River Laboratories). Experimental methods are described elsewhere [26]. Infections in experiment 1 were started with a mixed inoculum of ~10 resistant parasites and 10^6 susceptible parasites and were also either left untreated, or treated with pyrimethamine beginning on day 6 PI. Mice in one half of experiment 2 were inoculated with 10^6 susceptible parasites on day 0 and subsequently received ~25 resistant parasites five days later, the remaining control mice were sham-injected with uninfected blood on day 0 and subsequently inoculated with ~25 resistant parasites on day 5. Such extremely unequal starting conditions were chosen to generate what we knew from our previous work would be considerable (experiment 1) and overwhelming (experiment 2) competitive suppression of resistant parasites in the absence of chemotherapy [26–28]. Subsequently, these mice were either left untreated, or treated with an aggressive, moderate or light treatment of pyrimethamine one day later (on day 6 post susceptible infection). The pyrimethamine dosages and number of mice in each treatment group of each experiment are given in Table 1. Mice in experiment 1 were sampled daily up to day 21 and three times a week thereafter until day 49. Mice in experiment 2 were sampled daily up to day 28, with gametocyte densities measured from day 10 onwards. Weight, red blood cell density, asexual parasite density (using qPCR) and gametocyte density (using RT-qPCR) of both clones were estimated throughout the course of infection as shown elsewhere [26] with the exception of asexual parasite density estimations in experiment 2, for which the *CG1* assay was used [as in 28] instead of the *ama-1* assay.

Statistical analysis

The geometric mean parasite density over the whole infection period was calculated for clone R. As a measure of transmission potential, the predicted infectiousness was calculated for clone R from day 10 onwards using gametocyte densities in the density-infectivity function for clone R as derived from previous infection experiments [26] which relates gametocyte concentration to parasite prevalence in infected mosquitoes. These probabilities were integrated over time to give predicted proportion of mosquitoes infected over the course of the infection, assuming constant biting rate per day and no change in infectivity over time. Similarly, the overall infectiousness was calculated by combining both the resistant and susceptible gametocyte densities. Additionally, the length of the transmission period of the resistant parasites

was estimated by counting the number of gametocyte positive days for each infection. All analyses were done using analysis of variance in R 2.11.1 on mixed infections only. As explanatory variables, *drug treatment regime* (aggressive/moderate/light) was used. For overall infectiousness, also untreated infections were included.

Several mice were excluded from the analyses due to preterm death or failure to establish an infection. *Experiment 1*: Six mice failed to become infected with clone R (determined with the absence of detection by qPCR during the entire course of infection), presumably as a result of stochastic loss due to the low inoculum size (but note that, particularly in the untreated infections, there is a probability that this was a result from intense competitive suppression. To be conservative, failure of infection was assumed). *Experiment 2*: Seven mice died or were euthanized during the course of the infection (from mixed infections: three in untreated group, one in light treatment group, and one in moderate treatment group; of single clone R infections: one in light treatment group, one in moderate treatment group). Kinetics of infections in all mice is shown in Figs. S2 and S3 (Exp 1) and S4 and S5 (Exp 2).

Supporting Information

Figure S1 Parasite dynamics of resistant parasites in single clone infections of experiment 2 following aggressive treatment (dark blue line), moderate treatment (orange line), light treatment (green line) and untreated infections (light blue line). Different drug treatments do not affect the parasite dynamics. Sample sizes as in Table 1.
(EPS)

Figure S2 Asexual parasite dynamics of individual mice in experiment 1 with mixed infections of susceptible (black lines) and resistant (red lines) parasites in untreated infections (A–H), and infections that received a light (I–P), moderate (Q–X) and aggressive (Y-AF) drug treatment (for details, see Table 1). Grey areas show timing and duration of treatment. Asterisks indicate mice that were suspected of not having received resistant parasites as a result of stochastic loss due to low inoculation size and were excluded from the analysis (Table 1).
(EPS)

Figure S3 Gametocyte dynamics of individual mice in experiment 1 with mixed infections of susceptible (black lines) and resistant (red lines) parasites in untreated infections (A–H), and infections that received a light (I–P), moderate (Q–X) and aggressive (Y-AF) drug treatment (for details, see Table 1). Grey areas show timing and duration of treatment. Asterisks indicate mice that were suspected of not having received resistant parasites as a result of stochastic loss due to low inoculation size and were excluded from the analysis (Table 1).
(EPS)

Figure S4 Asexual parasite dynamics of individual mice in experiment 2 with mixed infections of susceptible (black lines) and resistant (red lines) parasites in untreated infections (A–J), and infections that received a light (K–T), moderate (U-AD) and aggressive (AE-AN) drug treatment (for details, see Table 1). Grey areas show timing and duration of treatment. Crosses indicate mice that died or were euthanized during the infection, asterisks indicate mice that had a lower parasite inoculum than intended. These mice were excluded from the analysis (Table 1).
(EPS)

Figure S5 Gametocyte dynamics of individual mice in experiment 2 with mixed infections of susceptible (black lines) and resistant (red lines) parasites in untreated infections (A–J), and infections that received a light (K–T), moderate (U-AD) and aggressive (AE-AN) drug treatment (for details, see Table 1). Grey areas show timing and duration of treatment. Crosses indicate mice that died or were euthanized during the infection, asterisks indicate mice that had a lower parasite inoculum than intended. These mice were excluded from the analysis (Table 1).
(EPS)

Figure S6 Mean asexual parasite dynamics (A,B) and gametocyte dynamics (C,D) of the susceptible clone under aggressive (dark blue lines), moderate (orange line), light (green line) or no treatment (light blue line) for experiment 1 (left panels) and experiment 2 (right panels). Data are means (± s.e.m.) with sample sizes as in Table 1. Note experiment 1 and 2 have a different duration of the experiment.
(EPS)

Figure S7 Mean total gametocyte dynamics (susceptible plus resistant gametocytes) in mixed infections under aggressive (dark blue lines), moderate (orange lines), light (green lines) or no treatment (light blue lines) for experiment 1 (A) and experiment 2 (B). Data are means (± s.e.m.) with sample sizes as in Table 1. Note experiment 1 and 2 have a different duration of experiment.
(EPS)

Figure S8 Probability of infection from the susceptible clone gametocyte density for experiment 1 (A) and experiment 2 (B) based on an established gametocyte density – infectivity relationship (see Materials and Methods) during the relapse period (day 10–28 PI). Data are means (± s.e.m.) with sample sizes as in Table 1.
(EPS)

Figure S9 Drug regimen model simulations (left panels) and experimental data from experiment 2 (right panels) of mixed infections of susceptible (black lines) and resistant (red lines) parasites under aggressive (A,B), moderate (C,D) and light (E,F) treatment regimens. Data in right-hand panels are means (± s.e.m.) with sample sizes as in Table 1. Grey areas demonstrate the timing and duration treatment. Of note is that the model, in contrast to the experimental mice, did not incorporate an immune function to control parasite densities in the later stages of the infection.
(EPS)

Acknowledgments

For discussion, we thank members of the Read-Thomas lab group, and the Research and Policy in Infectious Disease Dynamics program of the Science and Technology Directorate, Department of Homeland Security, and the Fogarty International Center, National Institutes of Health, particularly those at the Princeton RAPIDD workshop organized by J. Metcalf and R. Kouyos. We thank D. Kennedy,L. Pollitt and four anonymous reviewers for their comments on the MS.

Author Contributions

Conceived and designed the experiments: SH ASB NM AFR. Performed the experiments: SH ASB DGS DT. Analyzed the data: SH ASB. Wrote the paper: SH TD AFR. Performed within-host mathematical model: NM.

References

1. Fleming A (1964) Penicillin. Nobel Lectures, Physiology or Medicine 1942–1962. Amsterdam: Elsevier Publishing Company.
2. World Health Organization (2011) World malaria report 2011. Geneva, Switzerland. Available: http://www.who.int/malaria/world_malaria_report_2011.
3. World Health Organization (2012) The evolving threat of antimicrobial resistance: options for action. Available: http://whqlibdoc.who.int/publications/2012/9789241503181_eng.pdf
4. World Health Organization (2011) Global tuberculosis control: WHO report 2011. Available: http://www.who.int/tb/publications/global_report/2011/gtbr11_full.pdf
5. Deeks SG, Smith M, Holodniy M, Kahn JO (1997) HIV-1 protease inhibitors - A review for clinicians. J Am Med Assoc 277: 145–153.
6. Stratton CW (2003) Dead bugs don't mutate: Susceptibility issues in the emergence of bacterial resistance. Emerg Infect Dis 9: 10–16.
7. Martinez MN, Papich MG, Drusano GL (2012) Dosing regimen matters: the importance of early intervention and rapid attainment of the pharmacokinetic/pharmacodynamic target. Antimicrob Agents Chemother 56: 2795–2805.
8. Drusano GL, Liu W, Brown DL, Rice LB, Louie A (2009) Impact of short-course quinolone therapy on susceptible and resistant populations of *Staphylococcus aureus*. J Infect Dis 199: 219–226.
9. Drusano GL (2004) Antimicrobial pharmacodynamics: Critical interactions of 'bug and drug'. Nat Rev Microbiol 2: 289–300.
10. Condra JH, Emini EA (1997) Preventing HIV-1 drug resistance. Sci Med 4: 14–23.
11. Roberts JA, Kruger P, Paterson DL, Lipman J (2008) Antibiotic resistance - What's dosing got to do with it? Crit Care Med 36: 2433–2440.
12. zur Wiesch PA, Kouyos R, Engelstadter J, Regoes RR, Bonhoeffer S (2011) Population biological principles of drug-resistance evolution in infectious diseases. Lancet Infect Dis 11: 236–247.
13. Rapoff MA (2010) Adherence to pediatric medical regimens, Second Edition. Dordrecht: Springer. 231 p.
14. Crow JF, Kimura M (1970) An introduction to population genetics theory. New York: Harper and Row. 591 p.
15. Read AF, Day T, Huijben S (2011) The evolution of drug resistance and the curious orthodoxy of aggressive chemotherapy. Proc Natl Acad Sci USA 108: 10871–10877.
16. de Roode JC, Culleton R, Bell AS, Read AF (2004) Competitive release of drug resistance following drug treatment of mixed *Plasmodium chabaudi* infections. Malar J 3: 33.
17. Gerrish PJ, Lenski RE (1998) The fate of competing beneficial mutations in an asexual population. Genetica 102–3: 127–144.
18. Miralles R, Gerrish PJ, Moya A, Elena SF (1999) Clonal interference and the evolution of RNA viruses. Science 285: 1745–1747.
19. Read AF, Taylor LH (2001) The ecology of genetically diverse infections. Science 292: 1099–1102.
20. Hastings IM (1997) A model for the origins and spread of drug-resistant malaria. Parasitology 115: 133–141.
21. Mackinnon MJ, Hastings IM (1998) The evolution of multiple drug resistance in malaria parasites. Trans R Soc Trop Med Hyg 92: 188–195.
22. Hastings IM, D'Alessandro U (2000) Modelling a predictable disaster: The rise and spread of drug-resistant malaria. Parasitol Today 16: 340–347.
23. Hastings IM (2006) Complex dynamics and stability of resistance to antimalarial drugs. Parasitology 132: 615–624.
24. Mackinnon MJ (2005) Drug resistance models for malaria. Acta Trop 94: 207–217.
25. Lipsitch M, Samore MH (2002) Antimicrobial use and antimicrobial resistance: a population perspective. Emerg Infect Dis 8: 347–354.
26. Bell AS, Huijben S, Paaijmans KP, Sim DG, Chan BHK, et al. (2012) Enhanced transmission of drug-resistant parasites to mosquitoes following drug treatment in rodent malaria. PLoS One 7: e37172.
27. Wargo AR, Huijben S, de Roode JC, Shepherd J, Read AF (2007) Competitive release and facilitation of drug-resistant parasites after therapeutic chemotherapy in a rodent malaria model. Proc Natl Acad Sci USA 104: 19914–19919.
28. Huijben S, Nelson WA, Wargo AR, Sim DG, Read AF (2010) Chemotherapy, within-host ecology and the fitness of drug-resistant malaria parasites. Evolution 64: 2952–2968.
29. Geli P, Laxminarayan R, Dunne M, Smith DL (2012) "One-Size-Fits-All"? Optimizing treatment duration for bacterial infections. PLoS One 7: e29838.
30. Aarestrup F (2012) Get pigs off antibiotics. Nature 486: 465–466.
31. Scalera NM, File TM, Jr. (2007) How long should we treat community-acquired pneumonia? Curr Opin Infect Dis 20: 177–181.
32. el Moussaoui R, de Borgie C, van den Broek P, Hustinx WN, Bresser P, et al. (2006) Effectiveness of discontinuing antibiotic treatment after three days versus eight days in mild to moderate-severe community acquired pneumonia: randomised, double blind study. Br Med J 332: 1355–1358.
33. Kaki R, Elligsen M, Walker S, Simor A, Palmay L, et al. (2011) Impact of antimicrobial stewardship in critical care: a systematic review. J Antimicrob Chemother 66: 1223–1230.
34. Pugh R, Grant C, Cooke RPD, Dempsey G (2011) Short-course versus prolonged-course antibiotic therapy for hospital-acquired pneumonia in critically ill adults. Cochrane Database Syst Rev: Cd007577.
35. Karageorgopoulos DE, Valkimadi PE, Kapaskelis A, Rafailidis PI, Falagas ME (2009) Short versus long duration of antibiotic therapy for bacterial meningitis: a meta-analysis of randomised controlled trials in children. Arch Dis Child 94: 607–614.
36. Molyneux E, Nizami SQ, Saha S, Huu KT, Azam M, et al. (2011) 5 versus 10 days of treatment with ceftriaxone for bacterial meningitis in children: a double-blind randomised equivalence study. Lancet 377: 1837–1845.
37. Esposito S, Tagliabue C, Picciolli I, Semino M, Sabatini C, et al. (2011) Procalcitonin measurements for guiding antibiotic treatment in pediatric pneumonia. Respir Med 105: 1939–1945.
38. Lipsitch M, Levin BR (1997) The population dynamics of antimicrobial chemotherapy. Antimicrob Agents Chemother 41: 363–373.
39. Tam VH, Louie A, Deziel MR, Liu W, Drusano GL (2007) The relationship between quinolone exposures and resistance amplification is characterized by an inverted U: A new paradigm for optimizing pharmacodynamics to counterselect resistance. Antimicrob Agents Chemother 51: 744–747.
40. Drusano GL, Liu W, Fregeau C, Kulawy R, Louie A (2009) Differing effects of combination chemotherapy with meropenem and tobramycin on cell kill and suppression of resistance of wild-type pseudomonas aeruginosa PAO1 and its isogenic MexAB efflux pump-overexpressed mutant. Antimicrob Agents Chemother 53: 2266–2273.
41. Handel A, Margolis E, Levin BR (2009) Exploring the role of the immune response in preventing antibiotic resistance. J Theor Biol 256: 655–662.
42. Luciani F, Sisson SA, Jiang HL, Francis AR, Tanaka MM (2009) The epidemiological fitness cost of drug resistance in *Mycobacterium tuberculosis*. Proc Natl Acad Sci USA 106: 14711–14715.
43. Wootton JC, Feng XR, Ferdig MT, Cooper RA, Mu JB, et al. (2002) Genetic diversity and chloroquine selective sweeps in *Plasmodium falciparum*. Nature 418: 320–323.
44. Talisuna AO, Bloland P, D'Alessandro U (2004) History, dynamics, and public health importance of malaria parasite resistance. Clin Microbiol Rev 17: 235–254.
45. Gatenby RA (2009) A change of strategy in the war on cancer. Nature 459: 508–509.
46. Gatenby RA, Silva AS, Gillies RJ, Frieden BR (2009) Adaptive therapy. Cancer Res 69: 4894–4903.
47. Silva AS, Kam Y, Khin ZP, Minton SE, Gillies RJ, et al. (2012) Evolutionary approaches to prolong progression-free survival in breast cancer. Cancer Res 72: 6362–6370.
48. Hegreness M, Shoresh N, Damian D, Hartl D, Kishony R (2008) Accelerated evolution of resistance in multidrug environments. Proc Natl Acad Sci USA 105: 13977–13981.
49. Pena-Miller R, Laehnemann D, Jansen G, Fuentes-Hernadez A, Rosenstiel P, et al. (in press) When the most potent combination of antibiotics selects for the greatest bacterial load: the smile –frown transition. PLoS Biol 11: e1001540.
50. Mideo N, Barclay VC, Chan BHK, Savill NJ, Read AF, et al. (2008) Understanding and predicting strain-specific patterns of pathogenesis in the rodent malaria *Plasmodium chabaudi*. Am Nat 172: E214–E238.
51. White NJ (2008) Qinghaosu (Artemisinin): The price of success. Science 320: 330–334.
52. Schneider P, Chan BHK, Reece SE, Read AF (2008) Does the drug sensitivity of malaria parasites depend on their virulence? Malar J 7: 257.
